# Objective numeracy exacerbates framing effects from decision-making under political risk

**DOI:** 10.1038/s41598-024-61099-y

**Published:** 2024-05-07

**Authors:** Erin B. Fitz, Dominik A. Stecuła, Matthew P. Hitt, Kyle L. Saunders

**Affiliations:** 1 Department of Political Science, Colorado State University, Fort Collins, USA; 2 School of Communication and Department of Political Science, The Ohio State University, Columbus, USA

**Keywords:** Prospect theory, Numeracy, Framing effects, Risk, Decision-making, US congress, Political knowledge, Human behaviour, Psychology

## Abstract

While Prospect Theory helps to explain decision-making under risk, studies often base frames on hypothetical events and fail to acknowledge that many individuals lack the ability and motivation to engage in complex thinking. We use an original survey of US adults (*N* = 2813) to test Prospect Theory in the context of the May 2023 debt ceiling negotiations in the US Congress and assess whether objective numeracy moderates framing effects. We hypothesize and find evidence to suggest that most respondents are risk-averse to potential gains and risk-accepting to potential losses; however, high numerates are more risk-averse and risk-accepting to gains and losses, respectively, than low numerates. We also find that need for cognition interacts with numeracy to moderate framing effects for prospective losses, such that higher need for cognition attenuates risk-acceptance among low numerates and exacerbates risk-acceptance among high numerates. Our results are robust to a range of other covariates and in models accounting for the interaction between political knowledge and need for cognition, indicating joint moderating effects from two knowledge domains similarly conditioned by the desire to engage in effortful thinking. Our findings demonstrate that those who can understand and use objective information may remain subjectively persuaded by certain policy frames.

## Introduction

Americans vary widely in their relative consumption of political information and subsequent levels of political knowledge^1^. Similarly, citizens vary in their capacities to process complex policy information, especially when such information is presented numerically^2,3^. Prior work demonstrates that policy information can have persuasive effects on public opinion and decision-making^4,5^. Yet, quantitative policy information itself cannot be delivered wholly devoid of context and framing. Elite political actors can and do subjectively frame the causes, consequences, and potential solutions to pressing policy problems when presenting such information^6^. Individuals with high levels of knowledge and/or cognitive acumen may also be more susceptible to framing effects, as processing more easily discernible information is cognitively cheaper^7^. Taken together, this suggests that even those with greater domain- and/or context-specific knowledge may remain vulnerable to cognitive biases elicited by information that is carefully and intentionally crafted by goal-oriented political elites.

Still, evidence remains mixed with regard to what type(s) of messaging elicits framing effects and for whom framing effects occur^7^. A large corpus of evidence based on Prospect Theory (henceforth referred to as PT), for example, indicates that people—when tasked with choosing between options framed as gains or losses—are generally risk-averse with respect to potential gains and risk-accepting with respect to potential losses, relative to a reference point^8–10^. However, research on individual-differences emphasizes that framing can depend on one’s prior knowledge, attitudes, beliefs, and motivations^7^. Given the importance of (and difference between) prior information and willingness to engage in complex thinking, it is regrettable that few PT-oriented studies use frames based on actual events^11^ and only more recently has such research attempted to account for individual differences, e.g., numerical ability, that help to explain how and the extent to which people think about certain types of information^12^.

Thus, our primary motivations are to: (1) assess framing effects in the context of an actual and salient political event—in this case, the May 2023 debt ceiling negotiations in the US Congress, (2) examine whether objective numeracy, i.e., one’s ability to use and process numerical information^13,14^ moderates framing effects, and (3) test whether objective numeracy and the need for cognition, i.e., one's “tendency to engage in and enjoy effortful cognitive activity,” (p. 1870)^15,16^, interact to moderate framing effects.

Modeling our experimental design after Tversky and Kahneman’s “Asian disease problem”^9^, we hypothesize and find evidence to suggest that while most respondents in our sample are risk-averse with respect to potential gains and risk-accepting with respect to potential losses, high numerates are more risk-averse and risk-accepting to potential gains and losses, respectively, compared to low numerates. We also find that objective numeracy and the need for cognition interact to moderate framing effects in the domain of losses, such that higher need for cognition attenuates risk-acceptance among low numerates but exacerbates risk-acceptance among high numerates. This interaction also jointly moderates framing effects with that for political knowledge and need for cognition, demonstrating the potential impact of two knowledge domains similarly conditioned by the motivation to engage in effortful thinking. In turn, our findings add to evidence on how people think about complex political issues by demonstrating that even those who can better understand and use objective information may remain subjectively persuaded by certain policy frames.

## Prospect theory and objective numeracy

As Tversky and Kahneman described, “Veridical perception requires that the perceived relative height of two neighboring mountains, say, should not reverse with changes of vantage point. Similarly, rational choice requires that the preference between options should not reverse with changes of frame," (p. 453)^9^. However, as their seminal work on PT^8^ demonstrated, decision making is often contingent upon framing, i.e., “when (often small) changes in the presentation of an issue or event produce (sometimes large) changes of opinion” (p. 104)^7^.

PT contrasts with the classical *homo economicus* expected utility hypothesis by providing a descriptive, rather than normative, theory of decision-making that allows for the violation of invariance caused by framing^17^. According to PT, the value of an outcome is a product of a decision weight, not strictly its probability, such that “low probabilities are overweighted, moderate and high probabilities are underweighted, and the latter effect is more pronounced than the former” (p. 456)^9^. In turn, when tasked to choose between a “certain” Decision A and a “risky” Decision B, decision-makers will generally be risk-averse regarding potential gains and risk-accepting regarding potential losses^8,9^.

Although the tenets of PT are well-established and well-replicated^18,19^, most extant studies use hypothetical frames based on hypothetical issues. In turn, our first objective is to add to the relatively smaller body of literature demonstrating that the assumptions of PT hold in the context of actual events with real-world policy implications^11^—in this case, the May 2023 debt ceiling negotiations in the US Congress. We also echo those who noted that much of the extant literature on PT focuses on characteristics of the messaging, rather than characteristics of those who receive the messaging^20,21^ and that there exist individual and contextual differences in framing effects^7,22^. People’s propensity to use heuristics in complex information environments^23^ suggests that how people perceive and use certain messaging can depend on which considerations are most cognitively accessible^7,24^.

We propose that one factor with the potential to moderate framing effects is objective numeracy, i.e., one’s ability to understand and use numerical information, as assessed by a battery of questions that gauge respondents' understanding of basic mathematical concepts and probabilistic reasoning^13^. Objective numeracy is relevant not only to one's understanding of economic information conveyed in quantities (e.g., GDP, prices, inflation, and unemployment rates)^2^ but also for PT, which tasks subjects with choosing between “certain” and “risky” options framed as gains or losses^8,9^. Because how people perceive and use quantitative information can depend on the prior information they have about its value^2^, we expect to find that—when presented with options involving numerical information—one’s objective numeracy should determine which option they most prefer.

We further anticipate those with higher objective numeracy will exhibit stronger framing effects compared to those low in objective numeracy. Despite the assumption that higher numeracy can help to inform better judgment and decision-making^25^, evidence demonstrates that knowledge can increase heuristic processing, leading more knowledgeable individuals to selectively view and interpret information that conforms with their predispositions, rather than that which is necessarily comprehensive or correct^26,27^. For example, Peters et al.^14^ found that high numerates were more likely than low numerates to rate inferior bets as attractive, to express less negative and more clear feelings about their chance of winning, and to express more positive feelings about the amount won. In short, high numerates were better able to understand and use—but also derived more value and affect from—numerical information, compared to low numerates, sometimes resulting in worse, rather than better, judgment and decision-making^14^.

As for why objective numeracy can result in worse judgment and decision-making, evidence tends to center around two potential explanations: the first being that high numerates are more sensitive than low numerates to the difference between “impossible” and “possible” outcomes^9,14^, the second being that high numerates focus more on calculations than gist^12,28^. Either instance is one in which we would expect to observe more profound framing effects among high numerates, compared to low numerates, regardless of frame. With regard to the May 2023 debt ceiling negotiations in the US Congress, this is not to suggest that high numerates are apolitical, but rather that high numerates might incur fewer cognitive costs in bringing quantitative information to the top of one’s mind, compared to low numerates, and therefore focus more on quantitative information than other, non-compensatory heuristics^12^.

Thus, after confirming that most respondents are risk-averse with respect to potential gains and risk-accepting with respect to potential losses regarding the May 2023 debt ceiling negotiations in the US Congress, we hypothesize:

### H1

Objective numeracy moderates framing effects in the domain of gains, such that high numerates have a greater probability of choosing the “certain” Deal A over the “risky” Deal B compared to low numerates.

### H2

Objective numeracy moderates framing effects in the domain of losses, such that high numerates have a greater probability of choosing the “risky” Deal B over the “certain” Deal A compared to low numerates.

We further propose that the extent to which numeracy moderates framing effects is conditioned by one's need for cognition (henceforth referred to as NFCog), i.e., one's “tendency to engage in and enjoy effortful cognitive activity,” (p. 1870)^15,16^. As the notion of cognitive costliness would suggest, one's ability to understand and use quantitative information does not necessarily reflect one's motivation to engage in more deliberate thinking^29,30^. For example, Bruine de Bruin et al.^31^ found that the negative relationship between age and numeracy is conditioned by individuals' NFCog—that is, older individuals do not necessarily have lesser numerical ability than younger individuals, but do have a decreased willingness to engage in complex thinking.

While there is some evidence indicating those who engage in more deliberate thinking may be more likely to reach accurate conclusions^25^, other research suggests information processing remains subject to existing bias(es), especially when biases are sufficiently strong and more affectively-driven^26^. As such, while we might expect NFCog to attenuate framing effects among low numerates, who derive less value and affect from numerical information, we would expect NFCog to exacerbate framing effects among high numerates, who derive more value and affect from numerical information^14^. As such, we hypothesize:

### H3

Objective numeracy and NFCog interact to moderate framing effects, such that higher need for cognition attenuates framing effects among low numerates and exacerbates framing effects among high numerates.

## Methods

We fielded an original survey on May 27, 2023, following the announcement that the White House and Congressional Republicans had reached a deal to raise the debt ceiling. We fielded the survey online using Qualtrics and gathered a sample of 3,463 US adults via Lucid, an online sample provider widely used in similar research^32–34^. Lucid provides proprietary compensation to their panel members in exchange for survey participation and uses a quota system to provide samples that align with US Census benchmarks for respondents’ age, gender, race, ethnicity, and region^35^.

Respondents were first asked a series of demographic questions (i.e., age, income, race, ethnicity, gender, and religiosity) for which we control. Respondents then received a series of randomized questions assessing objective numeracy and NFCog, as well as a range of other social, political, and psychological factors associated with numeracy and framing (also for which we control, i.e., partisanship, political knowledge, need for closure, trust in government, interest, authoritarianism, and Big Five personality traits). Respondents also received one randomized attention check; we omitted those who provided incorrect responses to this pre-treatment attention check (*N* = 650) prior to conducting our analysis, yielding a total sample of *N* = 2813.

Following these questions, one-half of respondents were randomly assigned to receive the *Jobs Gained* frame; the other one-half of respondents received the *Jobs Lost* frame. All respondents were presented with the following question and were asked to choose between two, objectively equivalent options:

As you know, the U.S. government has reached its debt limit and is at risk of defaulting on its debt as of June 5, 2023. The White House and GOP negotiators have reached a compromise to raise the debt ceiling and avoid default, but Congress has yet to vote on this deal and the economic impact remains unknown.

Imagine that raising the debt ceiling is expected to affect 6 million jobs and you are tasked with deciding between two alternative deals. Which would you choose?

[*Jobs Gained Frame*] If Deal A is chosen, 2 million jobs will be preserved; if Deal B is chosen, there is a 1/3 probability that 6 million jobs will be preserved and 2/3 probability that no jobs will be preserved.

[*Jobs Lost Frame*] If Deal A is chosen, 4 million jobs will be lost; If Deal B is chosen, there is a 1/3 probability that no jobs will be lost and 2/3 probability that 6 million jobs will be lost.

We provide complete details on all measures in our analysis, as well as descriptive statistics for this sample, in the Survey Question Wording and Coding and Table SM1 in the Supplementary Material. This study was deemed exempt from formal review by the Institutional Review Board (IRB) at Colorado State University and was carried out in accordance with APSA’s Principles and Guidance on Human Subject Research. All participants provided informed consent.

## Who are the numerate?

Before proceeding to our results, it is instructive to first discuss who are the numerate in our sample. Consistent with previous literature^3,13^, less than half of the sample correctly assessed a probability or correctly converted a percentage to a proportion. While most respondents failed to correctly convert a proportion into a percentage or extrapolate the proportion of risk, most respondents did correctly assess the magnitude of risk. Most respondents also correctly converted a percentage to a proportion and a proportion to a percentage but failed to correctly convert a probability to a proportion. We show the percentage of correct responses for each question included in the 11-item objective numeracy battery, item and factor analyses indicating all 11 items fit well into a unidimensional scale, and the distribution of the 11-item numeracy scale (*M* = 6.58, *Mdn* = 7, *SD* = 2.86) in Table SM2 and Fig. SM1 in the Supplementary Materials.

After dichotomizing the objective numeracy scale via a median split (indicating low and high objective *Numeracy*, as is convention in extant numeracy literature^3,13,14^), we examine the predictors of objective *Numeracy* as it relates to our sample, shown in Table 1. Consistent with extant literature^3,36–40^, logistic regression results indicate *White* race, *Income*, *Education*, and *Political Knowledge* are positively associated with *Numeracy*; *Female* gender and *Authoritarianism* are negatively associated with *Numeracy.* Contrary to some previous evidence^31,36^, *Conscientiousness* and *Agreeableness* are positively associated with *Numeracy* and *NFCog* is negatively associated with *Numeracy*.

**Table 1:** Logistic regression results for numeracy.

	B	SE	p-value
NFCog	− 1.08	(0.39)	0.006
Age	0.09	(0.24)	0.698
White	0.45	(0.12)	0.000
Hispanic	− 0.47	(0.15)	0.001
Female	− 0.62	(0.09)	0.000
Income	0.86	(0.19)	0.000
Education	0.98	(0.21)	0.000
Religiosity	− 0.45	(0.13)	0.001
Party ID	0.08	(0.13)	0.562
Trust in Gov't	− 0.41	(0.18)	0.027
Political interest	− 0.31	(0.21)	0.135
Political knowledge	1.46	(0.15)	0.000
Need for closure	1.92	(0.37)	0.000
Authoritarianism	− 0.78	(0.15)	0.000
Openness	0.44	(0.28)	0.123
Conscientiousness	0.74	(0.25)	0.003
Extraversion	− 0.33	(0.19)	0.084
Agreeableness	0.56	(0.26)	0.031
Neuroticism	− 0.22	(0.23)	0.331
Constant	− 2.68	(0.47)	0.000
*N*	2710		

B denotes logistic regression coefficients; SE denotes standard errors.

Although previous scholarship accounted for numeracy alongside factors like the need for cognitive closure^33^, trust and race^40^, subjective values^41^, partisanship^27^, we add to extant literature by being the first, to our knowledge, to explicitly demonstrate that *Need for Closure* is positively associated with *Numeracy*, that *Hispanic* ethnicity and *Religiosity* are negatively associated with *Numeracy,* and that *Party ID* is not associated with *Numeracy* (nor is *Ideology*, which is strongly correlated with *Party ID* and yields virtually identical results when substituted for *Party ID* in all models in our analysis). Collectively, these findings help to further validate our sample and this measure by demonstrating that objective *Numeracy* has the potential to capture individual differences in how people process numerical information.

## Results

Turning to our main analysis, most respondents (67%) randomly assigned to the *Jobs Gained* frame (*M* = 0.67, *SD* = 0.47) selected the “certain” Deal A over the “risky” Deal B; in addition, most respondents (59%) who received the *Jobs Lost* frame (*M* = 0.59, *SD* = 0.49) selected the “risky” Deal B over the “certain” Deal A. In line with PT, this provides more evidence to suggest that people are generally risk-averse with respect to potential gains and risk-accepting with respect to potential losses. It also speaks to the relatively smaller body of research demonstrating that the assumptions of PT hold in the context of actual, as opposed to imagined, events.

Shifting our focus to individual differences, results from separate logistic regression models (with the modal response for each frame coded as “1” in each binary dependent variable) provide evidence in support of both hypotheses: In line with *H1*, *Numeracy* is positively associated with choosing the “certain” Deal A over the “risky” Deal B in the *Jobs Gained* frame. In line with *H2*, *Numeracy* is also positively associated with choosing the “risky” Deal B over the “certain” Deal A in the *Jobs Lost* frame. These results are consistent in bivariate analyses (Table 2, Models 1 and 2) and models accounting for a robust range of controls (Table 2, Models 3 and 4).

**Table 2:** The moderating effect of numeracy.

	(1)			(2)			(3)			(4)		
	Jobs gained frame			Jobs lost frame			Jobs gained frame			Jobs lost frame		
	B	SE	p-value	B	SE	p-value	B	SE	p-value	B	SE	p-value
Numeracy	0.49	(0.12)	0.000	0.50	(0.11)	0.000	0.44	(0.14)	0.001	0.28	(0.12)	0.023
NFCog							0.46	(0.53)	0.385	− 1.00	(0.48)	0.039
Age							0.06	(0.34)	0.850	0.44	(0.31)	0.156
White							− 0.18	(0.16)	0.246	− 0.21	(0.14)	0.152
Hispanic							0.09	(0.18)	0.637	− 0.28	(0.19)	0.129
Female							0.01	(0.13)	0.907	0.13	(0.12)	0.306
Income							− 0.29	(0.27)	0.270	− 0.12	(0.26)	0.648
Education							0.55	(0.29)	0.056	0.14	(0.28)	0.608
Religiosity							− 0.01	(0.19)	0.963	− 0.00	(0.18)	0.993
Party ID							− 0.25	(0.18)	0.159	− 0.21	(0.17)	0.227
Trust in Gov’t							0.17	(0.25)	0.496	− 0.41	(0.24)	0.086
Political Interest							− 0.26	(0.28)	0.362	0.12	(0.27)	0.655
Political Knowledge							0.08	(0.20)	0.678	0.61	(0.19)	0.002
Need for Closure							1.03	(0.45)	0.021	0.27	(0.46)	0.560
Authoritarianism							− 0.17	(0.20)	0.393	− 0.01	(0.19)	0.951
Openness							0.06	(0.37)	0.870	0.41	(0.37)	0.266
Conscientiousness							0.35	(0.34)	0.300	0.23	(0.32)	0.468
Extraversion							0.00	(0.26)	0.997	0.31	(0.25)	0.213
Agreeableness							0.55	(0.35)	0.117	-0.08	(0.33)	0.823
Neuroticism							0.42	(0.31)	0.179	0.13	(0.30)	0.663
Constant	0.50	(0.07)	0.000	0.15	(0.07)	0.037	− 1.23	(0.60)	0.040	− 0.18	(0.59)	0.764
AIC	1747.22			1906.15			1692.19			1834.67		
BIC	1757.69			1916.67			1801.31			1944.34		
*N*	1385			1419			1334			1370		

B denotes logistic regression coefficients; SE denotes standard errors.

**Figure 1:**
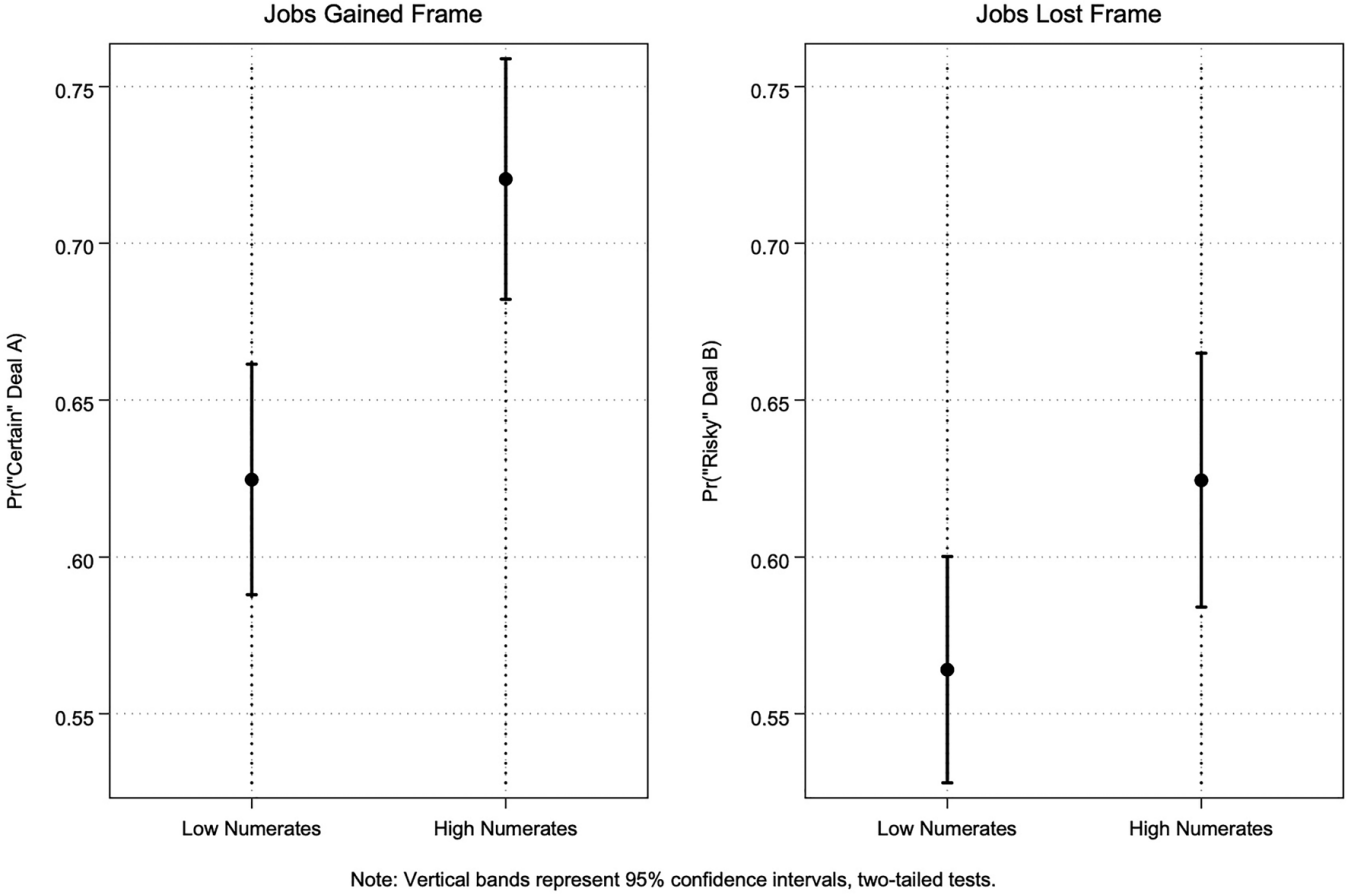
Predicted marginal effects for numeracy.

We illustrate the predicted marginal effects of *Numeracy* (based on results from Table 2, Models 3 and 4) in Fig. 1. These help to demonstrate that although both low and high numerates are associated with choosing the “certain” Deal A in the domain of gains and the “risky” Deal B in the domain of losses, as is expected according to PT, the predicted marginal effect of *Numeracy* is greater for high numerates than low numerates, regardless of frame (0.72 and 0.63 for high and low numerates, respectively, in the *Jobs Gained* frame; 0.63 and 0.56 for high and low numerates, respectively, in the *Jobs Lost* frame).

Regarding *H3*, the interaction term between *Numeracy* and *NFCog* is positively associated with choosing the modal response (i.e., the “risky” Deal B) in the *Jobs Lost* frame. Here again, these results are consistent in models without (Models 1 and 2) and with (Models 3 and 4) additional controls. Although *Numeracy* and *NFCog* do not interact to moderate the *Jobs Gained* frame in our main results (Table 3, Models 1 and 3), we find this unsurprising given a core assumption in PT is that “losses loom larger than gains” (p. 456)^9^.

**Table 3:** Numeracy × NFCog.

	(1)			(2)			(3)			(4)		
	Jobs gained frame			Jobs lost frame			Jobs gained frame			Jobs lost frame		
	B	SE	p-value	B	SE	p-value	B	SE	p-value	B	SE	p-value
Numeracy	0.65	(0.55)	0.231	− 1.14	(0.52)	0.028	0.68	(0.56)	0.224	− 1.17	(0.54)	0.029
NFCog	0.28	(0.57)	0.621	− 2.16	(0.51)	0.000	0.61	(0.63)	0.331	− 1.77	(0.56)	0.002
Numeracy × NFCog	− 0.31	(1.02)	0.763	3.05	(0.97)	0.002	− 0.47	(1.05)	0.655	2.79	(1.01)	0.006
Age							0.06	(0.34)	0.862	0.49	(0.31)	0.113
White							− 0.18	(0.16)	0.252	− 0.22	(0.15)	0.126
Hispanic							0.08	(0.18)	0.645	− 0.25	(0.19)	0.176
Female							0.02	(0.13)	0.899	0.15	(0.12)	0.236
Income							− 0.29	(0.27)	0.279	− 0.11	(0.26)	0.677
Education							0.55	(0.29)	0.055	0.12	(0.28)	0.658
Religiosity							− 0.01	(0.19)	0.960	0.01	(0.18)	0.942
Party ID							− 0.26	(0.18)	0.157	− 0.19	(0.17)	0.267
Trust in Gov’t							0.16	(0.25)	0.516	− 0.41	(0.24)	0.091
Political Interest							− 0.26	(0.28)	0.354	0.13	(0.27)	0.639
Political Knowledge							0.09	(0.21)	0.659	0.59	(0.20)	0.002
Need for Closure							1.04	(0.45)	0.020	0.19	(0.46)	0.673
Authoritarianism							− 0.17	(0.20)	0.385	0.01	(0.20)	0.951
Openness							0.06	(0.37)	0.880	0.50	(0.37)	0.180
Conscientiousness							0.35	(0.34)	0.302	0.22	(0.32)	0.489
Extraversion							0.00	(0.26)	0.987	0.29	(0.25)	0.254
Agreeableness							0.54	(0.35)	0.118	− 0.07	(0.34)	0.828
Neuroticism							0.42	(0.31)	0.176	0.07	(0.31)	0.806
Constant	0.34	(0.32)	0.277	1.33	(0.29)	0.000	− 1.32	(0.63)	0.036	0.26	(0.61)	0.672
AIC	1750.97			1890.09			1693.99			1828.94		
BIC	1771.91			1911.12			1808.30			1943.84		
*N*	1385			1419			1334			1370		

B denotes logistic regression coefficients; SE denotes standard errors.

**Figure 2:**
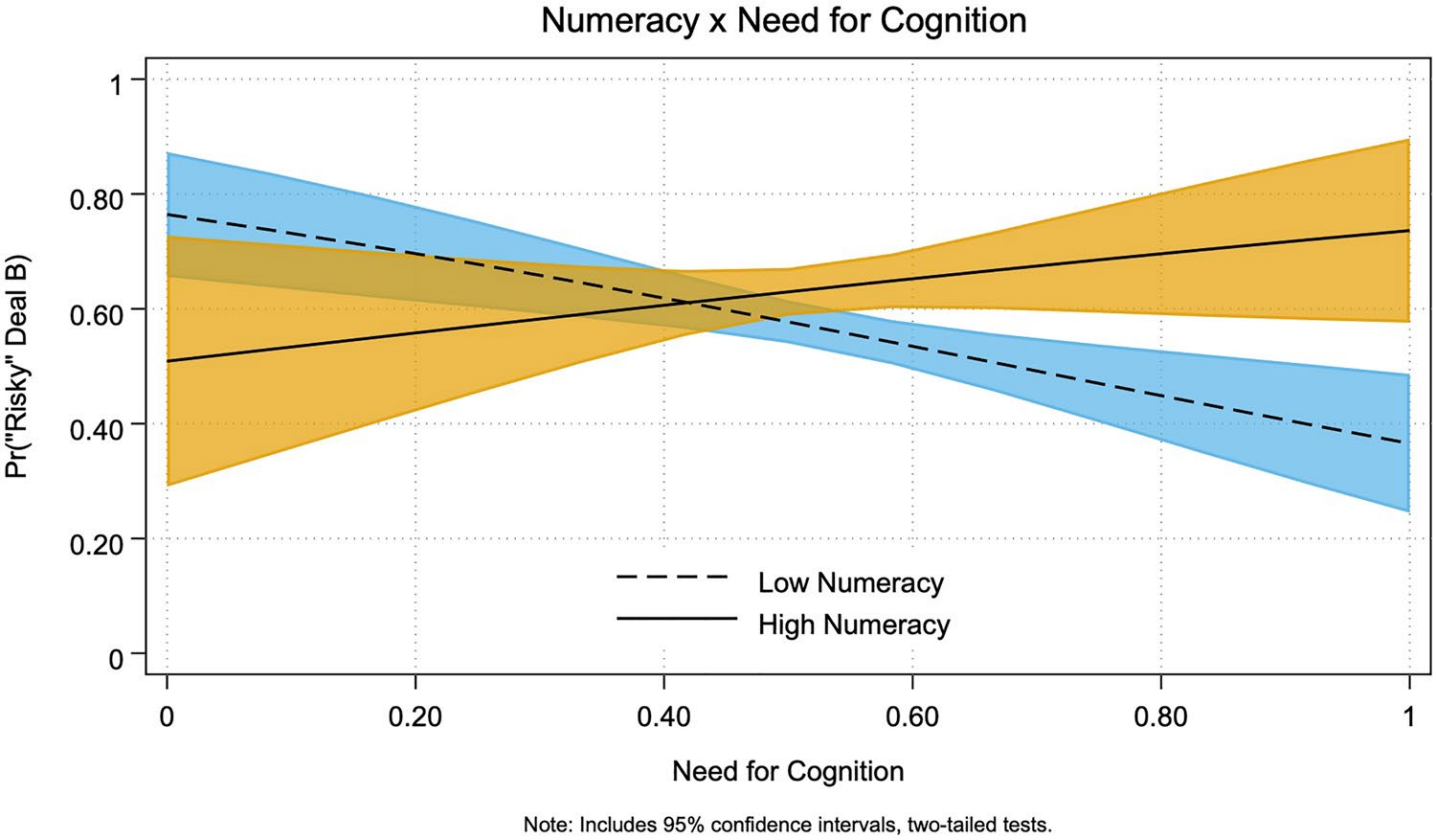
Marginal effects for numeracy × NFCog.

We illustrate the predicted marginal effects of *Numeracy* x *NFCog* (based on results from Table 3, Model 4) in Fig. 2. As expected, higher *NFCog* attenuates risk-acceptance among low-numerates (with marginal effects ranging from 0.76 to 0.37) and exacerbates risk-acceptance among high numerates (with marginal effects ranging from 0.51 to 0.74).

## How does objective numeracy compare to political knowledge?

We hypothesized and found evidence to suggest that objective *Numeracy* (and its interaction with *NFCog*) moderates framing effects such that high numerates have a greater probability of choosing the modal response regardless of frame. Yet, whether and the extent to which *Numeracy* is like other domain-specific knowledge remains an open question. For example, previous work found objective numeracy is distinct from general intelligence^14^ and other numerical competencies^42^; high numerates may also be more likely than low numerates to selectively interpret attitude-congenial quantitative information^27^. Much like the idea that one’s ability to engage in a certain type of thinking is not necessarily indicative of one’s motivation to do so^29,30^, the implication of extant numeracy literature is that we cannot be sure which heuristic one will rely on amidst decision-making under risk, especially when frames elicit multiple, sometimes competing, knowledge domains.

In turn, we follow previous numeracy scholarship by supplementing our main findings with additional tests that examine the effects of *Numeracy* when pitted against other relevant knowledge domains. Given our use of *Realpolitik* frames on the May 2023 US debt ceiling debates in US Congress, we would expect that, in addition to objective *Numeracy*, *Political Knowledge* (assessed with a series of six questions on the political system and US political issues; see the Survey Question Wording and Coding and Fig. SM2 in the Supplementary Materials for complete details) might also play a role in decision-making under risk. Previous results using *Political Knowledge* as a control variable support this assumption, indicating that despite the positive association between *Numeracy* and *Political Knowledge* (see Table 1), both factors remain positively associated with choosing the modal, risk-accepting response in the *Jobs Lost* frame (see Model 4 in Tables 2 and 3).

As such, two questions remain: Do *Political Knowledge* and *NFCog* similarly interact to moderate framing effects, and, if so, does this effect complement or mitigate that of *Numeracy* and its interaction with *NFCog*? Similar to previous literature indicating high numerates can better understand and use, but also derive more value and affect from, numerical information^14,42^, evidence on political sophistication also finds that expertise can lead to better decision-making; however, the advantage “is not that they have a stupendous amount of knowledge, but that they know how to get the most out of the knowledge they do possess”^24,43^. Along these lines, *NFCog* reflects certain individuals’ “strong motivation to understand the information that they process” (26)^16,44^ but, increasing information accessibility can subsequently increase bias^45^. Taken together, we would expect to find that *NFCog* has the potential to interact with virtually any type of domain-specific knowledge elicited by framing, including *Political Knowledge* for politically themed frames. Given numerical ability can aid in, but is not limited to, the interpretation political information, we expect our data to have captured both potential knowledge domains—one for quantitative information and one for political information—that are similarly conditioned by one’s motivation to engage in complex thinking.

We test these assumptions using an additional set of logistic regression models, shown in Table 4. Adding on to the last set of models in Table 3 that include all controls, results from Models 1 and 2 in Table 4 align with our expectation that *Political Knowledge* and *NFCog* interact to moderate decision-making in the *Jobs Lost* frame. Results from a complete pairwise-interaction model^46^ in Models 3 and 4 further indicate both interaction terms of interest (*Numeracy* × *NFCog* and *Political Knowledge* × *NFCog)* jointly moderate framing effects in the *Jobs Lost* frame; however, *Numeracy* and *Political Knowledge* do not interact with each other to moderate either frame (to illustrate that our results were not merely an artifact of our modeling strategy, we also show results from these models without additional controls in Table SM3 of the Supplementary Materials).

**Table 4:** Political knowledge × NFCog and complete pairwise-interaction model.

	(1)			(2)			(3)			(4)		
	Jobs gained frame			Jobs lost frame			Jobs gained frame			Jobs lost frame		
	B	SE	p-value	B	SE	p-value	B	SE	p-value	B	SE	p-value
Political knowledge	1.34	(0.83)	0.107	− 1.64	(0.79)	0.038	1.49	(0.90)	0.099	− 1.21	(0.83)	0.146
Numeracy	0.45	(0.14)	0.001	0.27	(0.12)	0.029	0.55	(0.62)	0.379	− 0.81	(0.59)	0.171
NFCog	1.66	(0.94)	0.076	− 2.91	(0.82)	0.000	1.73	(0.95)	0.070	− 3.19	(0.84)	0.000
Political knowledge × NFCog	− 2.36	(1.51)	0.119	4.21	(1.44)	0.003	− 2.48	(1.60)	0.121	3.47	(1.48)	0.019
Numeracy × NFCog							0.06	(1.10)	0.959	2.23	(1.04)	0.032
Numeracy × political knowledge							− 0.23	(0.40)	0.573	− 0.12	(0.37)	0.744
Age	0.04	(0.34)	0.900	0.50	(0.31)	0.108	0.05	(0.34)	0.875	0.53	(0.31)	0.088
White	− 0.18	(0.16)	0.245	− 0.19	(0.15)	0.189	− 0.19	(0.16)	0.233	− 0.21	(0.15)	0.154
Hispanic	0.08	(0.18)	0.645	− 0.27	(0.19)	0.145	0.08	(0.18)	0.647	− 0.25	(0.19)	0.185
Female	0.01	(0.13)	0.910	0.14	(0.12)	0.273	0.02	(0.13)	0.906	0.15	(0.12)	0.225
Income	− 0.29	(0.27)	0.273	− 0.11	(0.26)	0.677	− 0.29	(0.27)	0.269	− 0.10	(0.26)	0.700
Education	0.56	(0.29)	0.053	0.12	(0.28)	0.654	0.57	(0.29)	0.049	0.11	(0.28)	0.683
Religiosity	− 0.01	(0.19)	0.939	0.00	(0.18)	0.981	− 0.02	(0.19)	0.928	0.02	(0.18)	0.929
Party ID	− 0.24	(0.18)	0.176	− 0.20	(0.17)	0.233	− 0.24	(0.18)	0.182	− 0.19	(0.17)	0.265
Trust in Gov’t	0.15	(0.25)	0.556	− 0.42	(0.24)	0.086	0.15	(0.25)	0.548	− 0.41	(0.24)	0.091
Political interest	− 0.27	(0.28)	0.341	0.15	(0.27)	0.581	− 0.27	(0.28)	0.346	0.15	(0.27)	0.581
Need for closure	1.08	(0.45)	0.016	0.36	(0.46)	0.442	1.08	(0.45)	0.016	0.29	(0.47)	0.538
Authoritarianism	− 0.17	(0.20)	0.377	0.01	(0.20)	0.945	− 0.18	(0.20)	0.370	0.03	(0.20)	0.893
Openness	0.01	(0.38)	0.968	0.50	(0.37)	0.177	0.02	(0.38)	0.968	0.56	(0.38)	0.135
Conscientiousness	0.34	(0.34)	0.315	0.21	(0.32)	0.514	0.34	(0.34)	0.313	0.21	(0.33)	0.525
Extraversion	0.02	(0.27)	0.948	0.26	(0.25)	0.307	0.02	(0.27)	0.954	0.25	(0.25)	0.326
Agreeableness	0.55	(0.35)	0.118	− 0.08	(0.34)	0.802	0.53	(0.35)	0.130	− 0.09	(0.34)	0.792
Neuroticism	0.44	(0.31)	0.159	0.12	(0.30)	0.695	0.44	(0.31)	0.155	0.08	(0.31)	0.799
Constant	− 1.89	(0.74)	0.010	0.73	(0.67)	0.277	− 1.96	(0.75)	0.009	0.89	(0.69)	0.196
AIC	1691.75			1827.90			1695.43			1827.15		
BIC	1806.06			1942.80			1820.13			1952.49		
*N*	1334			1370			1334			1370		

B denotes logistic regression coefficients; SE denotes standard errors.

**Figure 3:**
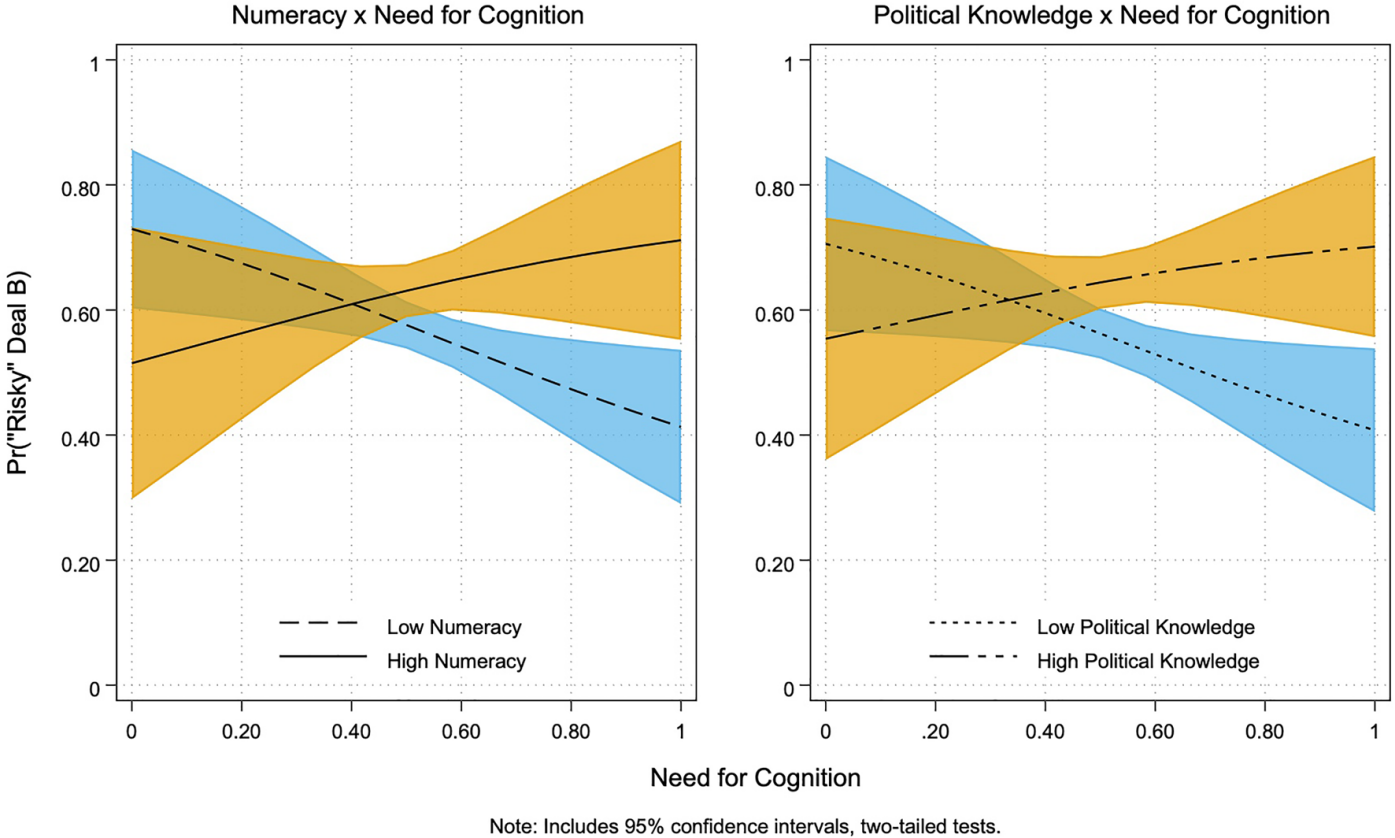
Marginal effects for political knowledge × NFCog.

For ease of interpretation, we run another set of models based on Table 4, Model 4, this time using a dichotomized measure of *Political Knowledge* via a median split. We then use these results (which are comparable to those in Table 4 and provided in Table SM4 in the Supplementary Materials) to plot predicted marginal effects. As illustrated in Fig. 3, the predicted probability of choosing the modal, risk-accepting Deal B in the *Jobs Lost* frame, when accounting for all control variables and additional terms in the complete pairwise-interaction model^46^, ranges from 0.73 to 0.41 for low *Numeracy,* 0.52 to 0.71 for high *Numeracy*, 0.71 to 0.41 for low *Political Knowledge,* and 0.55 to 0.70 for high *Political Knowledge*.

## Discussion

To explore how and for whom framing affects decision-making in the context of a real-world event, we test whether objective numeracy, i.e., one’s ability to understand and use numerical information, moderates decision-making in the context of the May 2023 debt ceiling debates in the US Congress. In line with Prospect Theory^8,9^, our results provide more evidence to suggest that people are generally risk-averse with respect to potential gains and risk-accepting with respect to potential losses. We also add to prior research on objective numeracy by demonstrating that high numerates–specifically, high need for cognition, high numerates—may be more strongly associated with paradoxical choices associated with persuasive frames^12^. Our findings are robust to a rigorous set of control variables associated with objective numeracy and framing, as well as models that account for the interaction between political knowledge and need for cognition, indicating a joint moderating effect of two knowledge domains similarly conditioned by one’s motivation to engage in effortful thinking. As such, these results help to bolster the validity of objective numeracy as a means to capture individual differences in the ability to process numerical information, as well as research that demonstrates numeracy does not attenuate, and may even exacerbate, existing bias^12^.

Yet, our study is not without limitations. Although our data, specifically with regard to the objective numeracy scale, are remarkably consistent with extant literature, we still cannot determine the exact mechanism behind what is objective numeracy and why it exacerbates framing effects. As previously mentioned, it may be that high numerates perceive a greater difference between “impossible” and “possible” outcomes^9,14^ or focus more on calculations than gist^12,28^. More specifically, high numerates may be able to broadly evaluate and use numerical information but cannot necessarily assess risk^13,14^. As Lipkus et al. described, adequately assessing risk requires “knowing not only the probabilities and magnitude of the risk but also how the risk magnitude compares to other hazards…how risk factors may modify one’s risk…and about the ease or difficulty of avoiding harm,” (p. 42)^13^. Given our results similarly suggest that numeracy “is only a small piece of a larger puzzle” (p. 42)^13^ that helps to explain decision-making under risk, we look forward to future scholarship that can further assess the validity of (and mechanisms behind) the objective numeracy scale.

We also acknowledge that, despite the robustness of our results, it remains to be seen whether objective numeracy and political knowledge (and their interactions with need for cognition) jointly moderate decision-making for other frames. Thus, we look forward to future scholarship that can better assess these and other types of knowledge (as well as the motivation to engage in complex thinking) in various contexts, including salient, real-world events. These areas, among others, remain important directions for future research given the assumption that many people are low in numeracy^18^ and the replication crisis in the social sciences.

As for what we make of these results, that these effects appear primarily in the *Jobs Lost* frame is perhaps unsurprising given the assumption that “losses loom larger than gains” (p. 456)^9^. However, they also raise the question of whether prospective losses, as opposed to prospective gains, prompt a stronger motivation to engage in complex thinking and what this motivation, when combined with the propensity to rely on heuristics in complex information environments^23^, means for the effectiveness of strategic frames. Considering the current state of crises and political polarization, we encourage scholars to continue exploration of how frames meant to elicit fear of a certain loss (the purported mechanism prompted by loss frames according to PT) might increase perceptions of politics as inherently decision-making under risk.

To conclude, our findings contribute to the debate surrounding what kind of information might best convey certain issues to the public by demonstrating that even those who can understand and use certain types of information may remain even more susceptible to cognitive biases^8,9^. In turn, the broader implication is that we cannot assume that any type of information is optimal from a normative perspective. Skilled political elites can effectively construct messages to conform with whatever their objective(s) may be^7^. Our work shows that efforts to increase knowledge in the mass public will not mitigate the efficacy of such strategies.

### Supplementary Information

Supplementary Information.

## Data Availability

Data and all supporting replication materials are publicly available at 10.7910/DVN/MMOGJI.
