# Supplementary material for: Objective numeracy exacerbates framing effects from decision-making under political risk
Source: Sci Rep. 2024 May 7;14:10473. doi: 10.1038/s41598-024-61099-y (PMC11076582; doi:10.1038/s41598-024-61099-y)
Supplement: Supplementary file 1 — Supplementary Information. [file 41598_2024_61099_MOESM1_ESM.pdf]

Supplementary Material for: Objective Numeracy Exacerbates Framing Effects from Decision-Making Under Political Risk

Erin B. Fitz[1], Dominik A. Stecuła[2], Matthew P. Hitt[3], and Kyle L. Saunders[4]

[1]Department of Political Science, Colorado State University
erin.fitz@colostate.edu, ORCiD 0000-0002-0780-0524
[2]Department of Political Science, Colorado State University
dominik.stecula@colostate.edu, ORCiD 0000-0001-6724-7559
[3]Department of Political Science, Colorado State University
matthew.hitt@colostate.edu, ORCiD 0000-0003-0762-1929
[4]Department of Political Science, Colorado State University
kyle.saunders@colostate.edu, ORCiD 0000-0003-4023-8729

Survey Question Wording and Coding 2

Table SM1: Descriptive Statistics 11

Table SM2: Proportion of Correct Responses for Items In the Objective Numeracy Scale with Item and Factor Analyses 12

Figure SM1: Distribution of 11-Item Numeracy Scale 13

Figure SM2: Distribution of Six-Item Political Knowledge Scale 14

Table SM3: Political Knowledge x NFCog (without controls) 15

Table SM4: Political Knowledge (Median Split) x NFCog 16

Survey Question Wording and Coding

Debt Ceiling. We assessed whether the tenets of Prospect Theory hold in the context of a real-world event by adapting questions from Tversky and Kahneman's[1] 'Asian disease problem' to fit into the context of the May 2023 debt ceiling negotiations in the US Congress:

As you know, the U.S. government has reached its debt limit and is at risk of defaulting on its debt as of June 5, 2023. The White House and GOP negotiators have reached a compromise to raise the debt ceiling and avoid default, but Congress has yet to vote on this deal and the economic impact remains unknown. Imagine that raising the debt ceiling is expected to affect 6 million jobs and you are tasked with deciding between two alternative deals. Which would you choose?

[*Jobs Gained Frame*] If Deal A is chosen, 2 million jobs will be preserved; If Deal B is chosen, there is a $\frac{1}{3}$ probability that 6 million jobs will be preserved and $\frac{2}{3}$ probability that no jobs will be preserved.

[*Jobs Lost Frame*] If Deal A is chosen, 4 million jobs will be lost; If Deal B is chosen, there is a $\frac{1}{3}$ probability that no jobs will be lost and $\frac{1}{3}$ probability that 6 million jobs will be lost.

We created a dummy variable for each set of responses, coded according to the expected modal response in Prospect Theory: For the Jobs Gained Frame, the dummy variable indicates whether respondents chose the 'sure thing' Deal A or the 'risky' Deal B (coded 1 and 0, respectively). For the Jobs Lost Frame, the dummy variable indicates whether respondents chose the 'sure thing' Deal A or the 'risky' Deal B (coded 0 and 1, respectively).

Numeracy. We assessed respondents' ability to understand and use numeric information using an 11-item objective numeracy scale[2]:

Imagine that we rolled a fair, six-sided die 1,000 times. Out of 1,000 rolls, how many times do you think the die would come up even (*2, 4,* or *6*)?

In the BIG BUCKS LOTTERY, the chances of winning a $10.00 prize are 1%. What is your best guess about how many people would win a $10.00 prize if 1,000 people each buy a single ticket from BIG BUCKS?

In the ACME PUBLISHING SWEEPSTAKE, the chance of winning a car is 1 in 1,000. What percent of tickets of ACME PUBLISHING SWEEPSTAKES win a car?

Which of the following numbers represents the biggest risk of getting a disease? *1 in 100, 1 in 1000, 1 in 10.*

Which of the following represents the biggest risk of getting a disease? *1%, 10%, 5%.*

If Person A's risk of getting a disease is 1% in ten years, and Person B's risk is double that of A's, what is B's risk?

If Person A's chance of getting a disease is 1 in 100 in ten years, and Person B's risk is double that of A, what is B's risk?

If the chance of getting a disease is 10%, how many people would be expected to get the disease out of 100?

If the chance of getting a disease is 10%, how many people would be expected to get the disease out of 1000?

If the chance of getting a disease is 20 out of 100, this would be the same thing as having a ____% chance of getting the disease.

The chance of getting a viral infection is .0005. Out of 10,000 people, about how many of them are expected to get infected?

We first created a dummy variable for each question to indicate whether respondents provided the incorrect or correct response for each question (coded 0 and 1, respectively). We also coded all non-responses as zero and note that tests wherein we excluded these responses did not substantively change our results. We then combined responses into an additive index ($\alpha = 0.80$) such that higher values indicate higher objective numeracy. Finally, as is common in other numeracy literature[3,4], we created a binary measure of objective numeracy, based on a median split of the additive index, to indicate low and high

objective *Numeracy* (coded 0 and 1, respectively).

Authoritarianism. We assessed respondents' level of authoritarianism, i.e., "a personality adaptation that values social cohesion and conformity to ingroup norms over personal freedom and individual autonomy,"[5] (540) using a battery of questions that tasks respondents with choosing between pairs of desirable qualities in children:

Although there are a number of qualities that people feel that children should have, every person thinks that some are more important than others. Below are pairs of desirable qualities. For each pair, please indicate which one you think is more important for a child to have. Independence or respect for elders, Curiosity or good manners, Obedience or self-reliance, Being considerate or well-behaved.

We coded responses for respect for elders, good manners, obedience, and well behaved as 1 and responses for independence, curiosity, self-reliance, and being considerate as 0. We combined these responses into a single measure ($\alpha = 0.59$) and recoded it to range from 0 to 1, such that higher values represent higher *Authoritarianism*.

Party Identification. We assessed respondents' partisan identity with a combination of two questions:

Generally speaking, do you usually think of yourself as *Republican*, a *Democrat*, an *Independent*, or *what*?

We then branched Democrats and Republicans into: Would you call yourself a *strong Democrat* [*strong Republican*] or a *not very strong Democrat* [*not very strong Republican*]? We branched Independents into: Do you think of yourself as closer to the *Democratic Party*, *Republican Party*, or *Neither*?

We combined responses to create a seven-point scale of *Party ID*, ranging from 1 = strong Democrat to 7 = strong Republican (with 4 = pure Independent) and recoded the measure to range from 0 to 1.

Ideology. We assessed respondents' self-reported ideology with the following question:

Here is a 7-point scale on which the political views that people might hold are arranged from extremely liberal to extremely conservative. Where would you place yourself on this scale? *Extremely liberal, Liberal, Slightly liberal, Moderate, middle of the road, Slightly conservative, Conservative, Extremely conservative.* We recoded *Ideology* to range from 0 to 1.

Political Knowledge. Because knowledge can help to inform policy judgments[6], we also asked respondents the following questions to assess their level of knowledge about politics:

Which job or political office is now held by Rishi Sunak? *Prime Minister of the United Kingdom, CEO of Target Corp., Prime Minister of Australia, Secretary of the Treasury.*

How many members are there in the U.S. House? *100, 50, 391, 435.*

Whose responsibility is it to nominate judges to U.S. federal courts? *The President, The House of Representatives, The Senate, The Supreme Court.*

How long is a term for a U.S. Senator? *2 years, 4 years, 6 years, 8 years.*

What job or political office does John Roberts currently hold? *Chair of the Democratic National Committee, Senate Majority Leader, Chief Justice of the Supreme Court, Chair of the Republican National Committee.*

What is the current U.S. national debt? *Between 5 trillion and 10 trillion U.S. dollars, Between 15 trillion and 20 trillion U.S. dollars, Between 30 trillion and 35 trillion U.S. dollars, Between 60 trillion and 65 trillion U.S. dollars.*

We recoded all correct responses (*Prime Minister of the United Kingdom, 435, The President, 6 years, Chief Justice of the Supreme Court*, and *Between 30 trillion and 35 trillion U.S. dollars*) as 1 and all incorrect responses as 0. We combined responses into a single measure ($\alpha = 0.75$) and recoded it to range from 0 to 1, such that higher values represent greater *Political Knowledge.*

Political Interest. We assessed respondents' political interest[7,8], both in the spe-

cific issue of the national debt limit and in politics more generally, with a series of three questions:

How closely are you following the debate over the national debt limit? Extremely closely, Very closely, Somewhat closely, Not very closely, Not closely at all.

How well would you say you understand the debate over increasing the national debt limit? *Extremely well, Very well, Somewhat well, Not very well, Not well at all.*

How often do you pay attention to what's going on in government and politics? *Always, Most of the time, About half the time, Some of the time, Never.*

We reverse coded responses to all three questions, combined these items into averaged scale ($\alpha = 0.87$), and recoded the measure to range from 0 to 1, such that higher values represent greater *Political Interest*.

Political Trust. We assessed respondents' level of political trust[19] with the following question:

How often can you trust the federal government in Washington to do what is right? Always, Most of the time, *About half the time, Some of the time, Never.* We recoded responses to range from 0 to 1.

Need for Closure. We assessed respondents' need for closure, i.e., "the expedient desire for any firm belief on a given topic, as opposed to confusion and uncertainty,"[10,11] (348) with the following 15-item battery, which asked respondents to indicate (on a seven-point scale ranging from Strongly disagree to Strongly agree) how much they disagree or agree with the following statements: 1) I don't like situations that are uncertain; 2) I dislike questions which could be answered in many different ways; 3) I find that a well-ordered life with regular hours suits my temperament; 4) I feel uncomfortable when I don't understand the reason why an event occurred in my life; 5) I feel irritated when one person disagrees with what everyone else in a group believes; 6) I don't like to go into a situation without knowing what I can expect from it; 7) When I have made a decision, I feel relieved; 8) When I am confronted with a problem, I'm dying to reach a solution very

quickly; 9) I would quickly become impatient and irritated if I would not find a solution to a problem immediately; 10) I don't like to be with people who are capable of unexpected actions; 11) I dislike it when a person's statement could mean many different things; 12) I find that establishing a consistent routine enables me to enjoy life more; 13) I enjoy having a clear and structured mode of life; 14) I do not usually consult many different opinions before forming my own view; and 15) I dislike unpredictable situations.

We averaged together all responses ($\alpha = 0.85$) and recoded the measure to range from 0 to 1, such that higher values represent greater *Need for Closure*.

Need for Cognition. We assessed the need for cognition, i.e., "a stable personality trait that describes individuals' tendency to engage in and enjoy effortful cognitive activity,"[12,13] (1870) using the six-item, NCS-6 scale, which asked respondents to indicate (on a six-point scale ranging from Strongly agree to Strongly disagree) how much they agree or disagree with the following statements: 1) I would prefer complex to simple problems; 2) I like to have the responsibility of handling a situation that requires a lot of thinking; 3) Thinking is not my idea of fun; 4) I would rather do something that requires little thought than something that is sure to challenge by thinking abilities; 5) I really enjoy a task that involves coming up with new solutions to problems; and 6) I would prefer a task that is intellectual, difficult, and important to one that is somewhat important but does not require much thought.

We reverse coded all items, combined all items into an averaged scale ($\alpha = 0.72$), and recoded the measure to range from 0 to 1, such that higher values represent greater *Need for Cognition*.

Big Five Personality Traits. We assessed respondents' Big Five personality traits[14] with a battery that asked to indicate (on a seven-point scale ranging from *Disagree strongly* to *Agree strongly*) the following:

Here are a number of personality traits that may or may not apply to you. Please indicate the extent to which you agree or disagree with each statement. You should rate

the extent to which the pair of traits applies to you, even if one characteristic applies more strongly than the other: 1) Extraverted, enthusiastic; 2) Critical, quarrelsome; 3) Dependable, self-disciplined; 4) Anxious, easily upset; 5) Open to new experiences, complex; 6) Reserved, quiet; 7) Sympathetic, warm; 8) Disorganized, careless; 9) Calm, emotionally stable; 10) Conventional, uncreative.

We reverse coded responses for *Reserved, quiet, Critical, quarrelsome, Disorganized, careless, Anxious, easily upset*, and *Conventional, uncreative*, then created the following variables out of five combined pairs: 1) *Extraversion*: [Extraverted, enthusiastic] and [Reserved, quiet]; 2) *Agreeableness*: [Critical, quarrelsome] and [Sympathetic, warm]; 3) *Conscientiousness*: [Dependable, self-disciplined] and [Disorganized, careless]; 4) *Neuroticism*: [Anxious, easily upset] and [Calm, emotionally stable]; and 5) *Openness*: [Open to new experiences, complex]and [Conventional, uncreative]. We recoded each variable to range from 0 to 1, such that higher values represent higher levels of each respective trait.

Education. What is your education level? *Did not complete HS, HS or GED, Some college, College graduate, Some postgraduate, Postgraduate degree.*

We recoded responses to range from 0 to 1, such that higher values represent higher levels of *Education.*

Income. What is, approximately, your current household annual income? *Under $20,000, $20,000-$39,999, $40,000-$59,000, $60,000-$79,999, $80,000-$99,999, $100,000-$124,999, $125,000-$149,999, $150,000-$199,999, $200,000 and over.*

We recoded responses to range from 0 to 1, such that higher values represent higher levels of Income.

Gender. What is your gender? *Female, Male, Other.*

We created an indicator variable indicating 0 = Not female and 1 = Female.

Age. What is your age?

We recoded respondents' self-reported Age (in years) to range from 0 to 1.

White. What is your race? *White, Black or African-American, American Indian*

or Alaska Native, Asian, Native Hawaiian or Other Pacific Islander, Some other race, I prefer not to answer.

We created a indicator variable indicating 0 = Not white and 1 = *White.*

Hispanic. Do you identify as Hispanic or Latino? *Yes, No.*

We created an indicator variable indicating 0 = Not Hispanic or Latino and 1 = *Hispanic* or Latino.

Religiosity. How would you classify your level of involvement with your religion or spirituality? *Very active, Moderately active, Neither active nor inactive, Moderately inactive, Very inactive.*

We reverse coded responses and recoded them to range from 0 to 1, such that higher values represent stronger *Religiosity.*

Attention Check. Most modern theories of decision making recognize that decisions do not take place in a vacuum. Individual preferences and knowledge, along with situational variables can greatly impact the decision process. To demonstrate that you've read this much, just go ahead and select both red and green among the alternatives below, no matter what your favorite color is. Yes, ignore the question below and select both of those options. What is your favorite color? *White, Black, Red, Pink, Green, Blue.*

We recoded incorrect responses for *White, Black, Pink*, and *Blue* as 0 and correct responses for *Red* and *Green* as 1.

References

1. Tversky, A. & Kahneman, D. The framing of decisions and the psychology of choice. *Science* 211, 453-458 (1981).

2. Lipkus, I. M., Samsa, G., & Rimer, B. K. General performance on a numeracy scale along highly educated samples. *Med. Decis. Mak.* **21**, 37-44 (2001).

3. Mèrola, V. & Hitt, M. P. Numeracy and the persuasive effect of policy information and party cues. *Public Opin Q.* **80**, 554-62 (2015).

4. Peters, E. *et al.* Numeracy and decision making. *Psychol. Sci.* **17**, 407–413 (2006).

5. Engelhardt, A. M., Feldman, S., & Hetherington, M. J. Advancing the measurement of authoritarianism. *Political Behav.* **45**, 537-560 (2021).

6. Lupia, A. *Uninformed: Why people seem to know so little about politics and what we can do about it* (Cambridge, 2015).

7. Miller, J. M. *et al.* Putting the political in political interest: The conditional effect of politics on citizens' interest in politics. *Am. Polit. Res.* **51**, 510-524 (2022).

8. Prior, M. *Hooked: How politics captures people's interest* (Cambridge, 2019)

9. Hetherington, M. J. *Why trust matters: Declining political trust and the demise of American liberalism* (Princeton, 2004)

10. Jost, J. T. *et al.* Political conservatism as motivated social cognition. *Psychol. Bull.* **129**, 339-375 (2003).

11. Kruglanski, A. W. & Webster, D. M. Motivated closing of the mind: "Seizing" and "freezing." *Psychol Rev.* **103**, 263-283 (1996).

12. Lins de Holanda Coelho, G., Hanel, P. H. P., & Wolf, L. J. The very efficient assessment of need for cognition: Developing a six-item version. *Assessment* **27**, 1870-85 (2020).

13. Cacioppo, J. T. & Petty, R. E. The need for cognition. *J. Pers. Soc. Psychol.* **42**, 116-131 (1982).

14. Costa, P. T. & McCrae, R. R. The five-factor model of personality and its relevance to personality disorders. *J. Pers. Disord.* **6**, 343-359 (1992).

Table SM1: Descriptive Statistics

	Obs	Mean	SD	Min	Max
Numeracy (Continuous)	2,813	6.58	2.86	0	11
Numeracy (Binary)	2,813	0.44	0.50	0	1
Age	2,798	0.35	0.21	0	1
White	2,775	0.77	0.42	0	1
Hispanic	2,798	0.12	0.33	0	1
Female	2,813	0.53	0.50	0	1
Income	2,747	0.31	0.25	0	1
Education	2,813	0.45	0.24	0	1
Party ID	2,795	0.47	0.36	0	1
Ideology	2,797	0.49	0.28	0	1
Political Knowledge	2,807	0.55	0.33	0	1
Need for Closure	2,812	0.78	0.13	0	1
Need for Cognition	2,812	0.53	0.13	0	1
Trust in Government	2,811	0.37	0.27	0	1
Political Interest	2,813	0.54	0.25	0	1
Religiosity	2,798	0.53	0.35	0	1
Authoritarianism	2,813	0.51	0.32	0	1
Openness	2,811	0.54	0.16	0	1
Conscientiousness	2,811	0.70	0.21	0	1
Extraversion	2,811	0.45	0.23	0	1
Agreeableness	2,811	0.67	0.20	0	1
Neuroticism	2,811	0.59	0.23	0	1

Table SM2: Proportion of Correct Responses for Items In the Objective Numeracy Scale with Item and Factor Analyses

	Question in 11-Item Objective Numeracy Scale	Proportion of Sample	Factor Loading
Q1	Imagine that we rolled a fair, six-sided die 1,000 times. Out of 1,000 rolls, how many times do you think the die would come up even (2, 4, or 6)? *Answer*: 500 out of 1000	0.48	0.50
Q2	In the BIG BUCKS LOTTERY, the chances of winning a $10.00 prize is 1%. What is your best guess about how many people would win a $10.00 prize if 1,000 people each buy a single ticket to BIG BUCKS? *Answer*: 10 persons out of 1000	0.64	0.47
Q3	In the ACME PUBLISHING SWEEPSTAKES, the chance of winning a car is 1 in 1,000. What percent of tickets to ACME PUBLISHING SWEEPSTAKES win a car? *Answer*: 0.1%	0.19	0.42
Q4	Which of the following numbers represents the biggest risk of getting a disease? 1 in 100; 1 in 1000; 1 in 10. *Answer*: 1 in 10	0.77	0.58
Q5	Which of the following numbers represents the biggest risk of getting a disease? 1%; 10%; 5%. *Answer*: 10%	0.87	0.37
Q6	If Person A's risk of getting a disease is 1% in ten years, and Person B's risk is double that of A's, what is B's risk? *Answer*: 2%	0.71	0.58
Q7	If Person A's chance of getting a disease is 1 in 100 in ten years, and person B's risk is double that of A's, what is B's risk? *Answer*: 2 out of 100	0.42	0.39
Q8	If the chance of getting a disease is 10%, how many people would be expected to get the disease: A: Out of 100? *Answer*: 10	0.75	0.76
Q9	If the chance of getting a disease is 10%, how many people would be expected to get the disease: B: Out of 1000? *Answer*: 100	0.69	0.67
Q10	If the chance of getting a disease is 20 out of 100, this would be the same as how a ____% chance of getting the disease. *Answer*: 20	0.67	0.63
Q11	The chance of getting a viral infection is .0005. Out of 10,000 people, how many of them are expected to get infected? *Answer*: 5 people	0.39	0.44
	Eigenvalue		3.24
	Proportion of shared variance		0.66
	Cronbach's alpha		0.80
	N		2,813

Figure SM1: Distribution of 11-Item Numeracy Scale

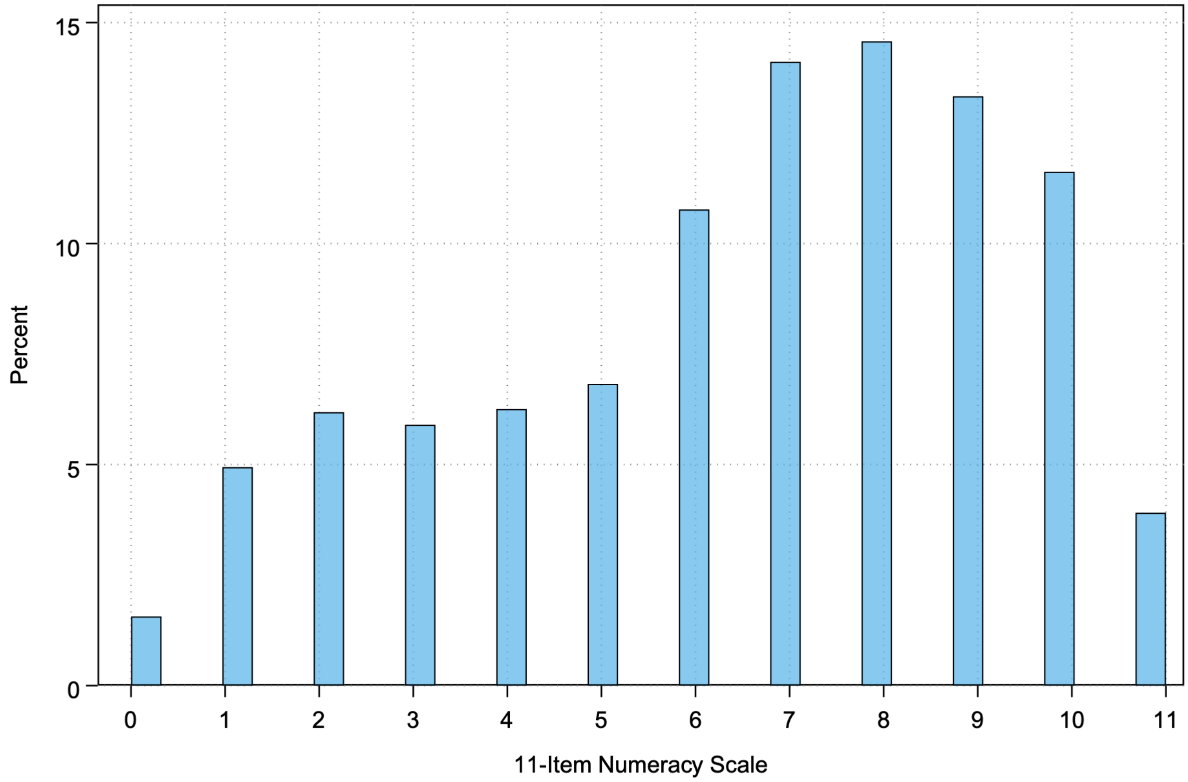

Figure SM2: Distribution of Six-Item Political Knowledge Scale

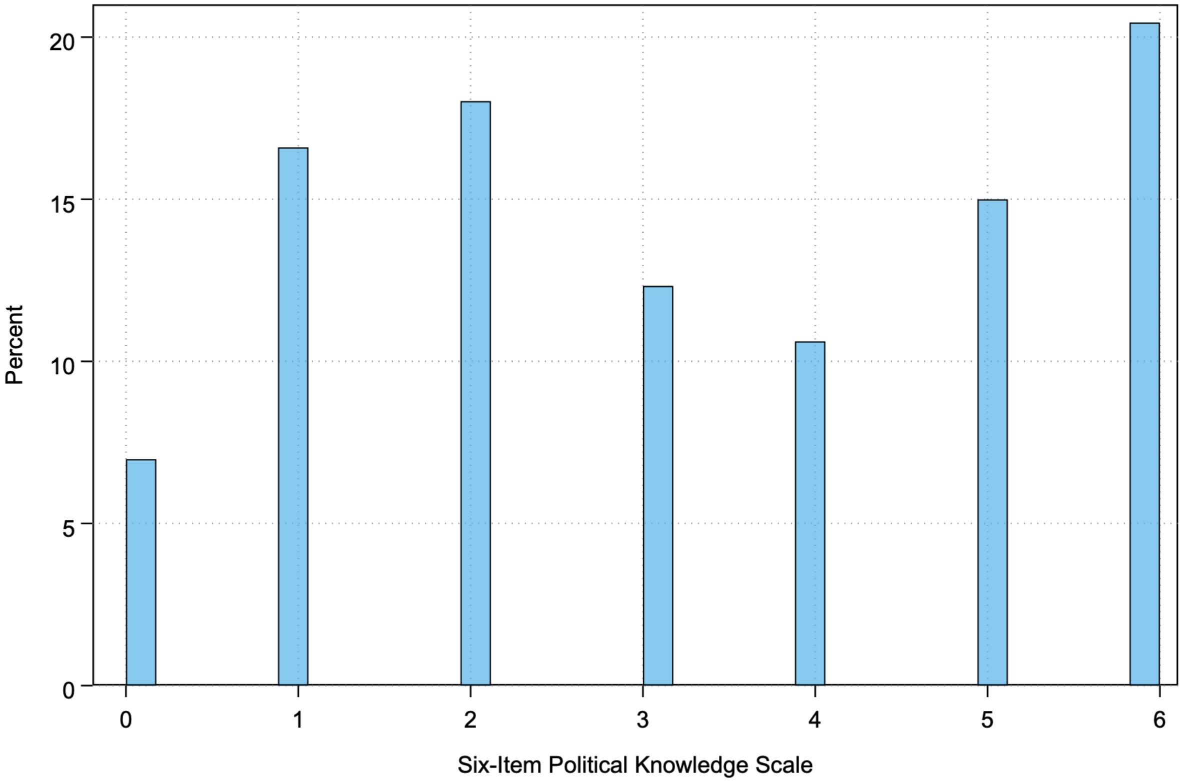

Table SM3: Political Knowledge x NFCog (without controls)

	(1)			(2)			(3)			(4)		
	Jobs Gained Frame			Jobs Lost Frame			Jobs Gained Frame			Jobs Lost Frame		
	B	SE	p-value	B	SE	p-value	B	SE	p-value	B	SE	p-value
Political Knowledge	0.31	(0.17)	0.070	0.93	(0.17)	0.000	1.28	(0.79)	0.107	-1.37	(0.77)	0.074
NFCog							0.90	(0.87)	0.299	-3.24	(0.78)	0.000
Political Knowledge x NFCog							-1.81	(1.45)	0.212	4.21	(1.41)	0.003
Constant	0.53	(0.11)	0.000	-0.14	(0.10)	0.171	0.04	(0.48)	0.931	1.63	(0.44)	0.000
AIC	1757.72			1893.09			1760.15			1878.11		
BIC	1768.18			1903.60			1781.08			1899.13		
N	1,382			1,418			1,382			1,418		

Note: B denotes logistic regression coefficients; SE denotes standard errors.

Table SM4: Political Knowledge (Median Split) x NFCog

	Jobs Lost Frame		
	B	SE	p-value
Political Knowledge (Median Split)	-0.72	(0.54)	0.185
Numeracy	-0.99	(0.56)	0.078
NFCog	-2.40	(0.64)	0.000
Political Knowledge x NFCog	2.10	(0.97)	0.030
Numeracy x NFCog	2.40	(1.03)	0.019
Numeracy x Political Knowledge	0.05	(0.24)	0.846
Age	0.50	(0.31)	0.111
White	-0.20	(0.15)	0.163
Hispanic	-0.28	(0.19)	0.132
Female	0.15	(0.12)	0.235
Income	-0.09	(0.26)	0.735
Education	0.11	(0.28)	0.695
Religiosity	0.01	(0.18)	0.960
Party ID	-0.19	(0.17)	0.262
Trust in Gov't	-0.40	(0.24)	0.098
Political Interest	0.14	(0.27)	0.616
Need for Closure	0.21	(0.46)	0.652
Authoritarianism	0.05	(0.20)	0.814
Openness	0.56	(0.38)	0.139
Conscientiousness	0.21	(0.32)	0.509
Extraversion	0.26	(0.25)	0.292
Agreeableness	-0.07	(0.34)	0.836
Neuroticism	0.06	(0.31)	0.854
Constant	0.69	(0.63)	0.272
AIC		1827.1	
BIC		1952.44	
N		1,370	

Note: B denotes logistic regression coefficients; SE denotes standard errors.
